# Severe Hypokalemia and Respiratory Muscle Paralysis: An Atypical Manifestation of Primary Sjögren’s Syndrome

**DOI:** 10.7759/cureus.76240

**Published:** 2024-12-23

**Authors:** Patrícia Sobrosa, Ângela Ferreira, Rita Vilar da Mota, Joana Couto, Luciana Sousa

**Affiliations:** 1 Internal Medicine, Hospital de Santa Luzia - Unidade Local de Saúde do Alto Minho, Viana do Castelo, PRT

**Keywords:** distal renal tubular acidosis, hypokalemia, respiratory muscle paralysis, sicca symptoms, sjögren’s syndrome

## Abstract

Primary Sjögren’s syndrome (SS) is a systemic autoimmune disorder primarily affecting exocrine glands, that may occasionally present with severe extra-glandular manifestations. Although rarely, severe hypokalemia and respiratory muscle paralysis may be initial presentations.

We report the case of a 33-year-old woman with a recent history of severe acute respiratory syndrome coronavirus 2 (SARS-CoV2) infection who presented with headache and generalized muscle weakness. An evaluation revealed severe hypokalemia, acute kidney injury, and metabolic acidosis. Despite intravenous potassium supplementation, her condition rapidly deteriorated, leading to respiratory failure requiring invasive ventilation. Further investigations showed a urinary pH >5.5, indicating distal renal tubular acidosis (dRTA). Positive antinuclear antibody (ANA) titers, SS-related antigen A antibody (anti-Ro/SSA), and lymphocytic infiltration on parotid gland biopsy confirmed a diagnosis of primary SS. Treatment with bicarbonate, potassium supplementation, and oral corticosteroids was initiated, leading to clinical improvement.

This case underscores the importance of considering SS in patients with unexplained dRTA and severe hypokalemia, emphasizing the importance of timely diagnosis and tailored therapy in managing systemic manifestations.

## Introduction

Sjögren’s syndrome (SS) is a chronic, slowly progressive multisystem autoimmune disease, characterized by lymphoproliferative features and mononuclear cell infiltration of exocrine glands [1,2]. It is heterogeneous in presentation, course, and outcome, lacking a single gold standard for diagnosis or classification [1]. The diagnosis of SS was established based on the 2016 American College of Rheumatology and European League Against Rheumatism (ACR-EULAR) classification criteria; reflecting the diverse manifestations of the disease, these criteria integrate clinical findings, serological markers, and histopathological evaluations, such as minor salivary gland biopsy findings.

The incidence of SS is estimated at three to 11 cases per 100000 individuals, with a prevalence of 0.1% to 4.8%, varying by ethnicity, sample size, and sex [2,3]. Diagnosis typically occurs between ages 30 and 60 but can occur across all age groups, predominantly affecting women with a female-to-male ratio of approximately 10:1 (a significantly higher disparity compared to other systemic autoimmune diseases) [2,4].

While SS primarily targets exocrine glands, particularly the lacrimal and salivary glands, it can also affect the upper respiratory tract, oropharynx, and in women, the vaginal mucosa [2]. Beyond these, SS can involve multiple extra-glandular systems, with cutaneous, articular, pulmonary, cardiovascular, nephro-urological, nervous, and hematological manifestations [2,4,5]. Extra-glandular symptoms may even precede classic sicca presentations [5]. The kidney is a particularly common site of extra-glandular involvement [6]. The hallmark of primary SS, reported by over 95% of patients, is mucosal dryness, particularly keratoconjunctivitis sicca, and xerostomia, which are independent of patient age or disease stage [2,4,5]. Systemic manifestations occur in approximately 30-40% of cases, though asymptomatic presentations are also possible [7].

Renal involvement in SS varies significantly, with a prevalence ranging from 18.45% to 67%, depending on the study design and diagnostic criteria [3]. Manifestations are heterogeneous, ranging from electrolyte imbalances to interstitial nephritis, glomerulonephritis, and distal renal tubular acidosis (dRTA), which is reported in 25-40% of the cases, indicating severe renal involvement [6,8].

Although uncommon, hypokalemic paralysis can be an initial presentation in approximately 7% of SS patients, highlighting the importance of clinical awareness [9].

We present a rare case of primary SS presenting with respiratory muscle paralysis due to severe hypokalemia. This report underscores the diagnostic process and clinical course that led to identifying SS as the underlying cause, emphasizing the need for timely recognition in cases with uncommon presentations.

## Case presentation

A 33-year-old woman with a history of depressive disorder presented to the Emergency Department (ED) with a 10-day history of headache and generalized muscle weakness. One week prior to symptom onset, she was diagnosed with a severe acute respiratory syndrome coronavirus 2 (SARS-CoV2) infection, which caused only mild upper respiratory symptoms that resolved with non-steroidal anti-inflammatory drugs (NSAIDs) (ibuprofen) and analgesics (paracetamol). In the ED, she was alert, oriented, and exhibited dysphonia and generalized proximal muscle weakness (grade 2/5) with hyporeflexia, without additional neurological abnormalities. 

Laboratory findings (Table 1) indicated acute kidney injury (creatinine 1.27 mg/dL) and hypokalemia (2.8 mmol/L). Intravenous (IV) fluid therapy with isotonic saline and potassium (K+) supplementation was initiated. Despite administering 3000 mg of potassium over six hours, her condition worsened, manifesting mental status alteration, progressive muscle paralysis, and respiratory distress with the need for orotracheal intubation. Arterial blood gas analysis demonstrated metabolic acidosis, decreased bicarbonate (HCO_3_^-^) levels, and severe hypokalemia (pH 7.02, HCO_3_^- ^7 mmol/L, pCO_2_ 27 mmol/L, K^+^ 1.1 mmol/L). Urinalysis showed a urinary pH >5.5 (measured at 7.5) and a positive urinary anion gap of 14, with urinary sodium of 76 mmol/L, potassium of 5 mmol/L, and chloride of 67 mmol/L. The serum anion gap was normal. Based on the clinical presentation (muscle paralysis) and laboratory findings suggestive of dRTA, further etiological investigation was pursued while continuing IV potassium and bicarbonate supplementation. Renal ultrasound showed no evidence of nephrocalcinosis or nephrolithiasis. Serological tests revealed positive antinuclear antibodies (ANA) (1:320, speckled pattern); positive SS-related antigen A antibody (anti-Ro/SSA); positive cyclic citrullinated peptide antibody (anti-CCP) (9.1 U/mL); and positive rheumatoid factor (RF) (191.3 IU/mL). 

**Table 1:** Arterial blood gas and laboratory test results ANA: antinuclear antibodies; ANCA: antineutrophil cytoplasmic antibodies; Anti-CCP: positive cyclic citrullinated peptide antibody; Anti-La/SSB: Sjögren’s syndrome-related antigen B antibody; Anti-Ro/SSA: Sjögren’s syndrome-related antigen A antibody; pCO_2_: partial pressure of carbon dioxide; RF: rheumatoid factor; HCO_3_^-^: bicarbonate; Cl^-^: chloride; K^+^: potassium; Na^+^: sodium; Ca^2+^: calcium; Mg^2+^: magnesium; PO_4_^3-^: phosphate

	Parameter	Patient Value	Reference Range	Units
Arterial blood gas	pH	7.02	7.35-7.45	
	HCO₃⁻	7	22-28	mmol/L
	pCO₂	27	35-45	mmHg
Biochemistry	Creatinine	1.27	0.6-1.2	mg/dL
	Na+	137	136-145	mmol/L
	K⁺	1.1	3.5-5.0	mmol/L
	Cl⁻	127	98-107	mmol/L
	Ca^2+^ corrected to albumin	7.9	8.6-10.3	mg/dL
	PO_4_^3-^	3.2	2.5-4.9	mg/dL
	Mg^2+^	2.6	1.6-2.6	mg/dL
	Albumin	3.4	3.5-5	g/dL
	Osmolality	317	280-301	mOsmol/kg
	Serum anion gap	16		
Urinalysis	pH	7.5	<5.5	
	Na⁺	76	<20	mmol/L
	K⁺	5		mmol/L
	Cl⁻	67	<40	mmol/L
	Urinary anion gap	14		
Serological tests	ANA	1:320 (speckled)		
	ANCA	Negative		
	Anti-Ro/SSA	Positive (+++)		
	Anti-La/SSB	Negative		
	RF	191.3	<15	IU/mL
	Anti-CCP	9.1	<5	U/mL

**Figure 1:**
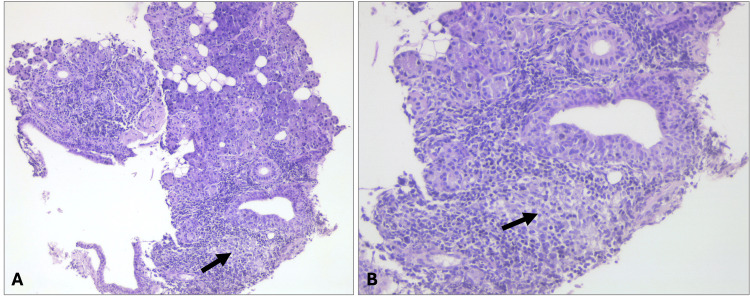
Parotid gland biopsy x10 (A) and x20 (B) revealed lymphoid aggregates with germinal centers infiltrating ductal and acinar structures (black arrows)

The patient’s condition improved following high-dose IV potassium and bicarbonate supplementation, enabling successful weaning from mechanical ventilation. Upon further evaluation, she reported a six-month history of asthenia and dry mouth. The clinical presentation, along with serological findings, was consistent with SS affecting the salivary glands and kidneys. A parotid gland biopsy revealed lymphoid aggregates with germinal centers infiltrating ductal and acinar structures (Figure 1).

A Schirmer test confirmed reduced tear production, further supporting the diagnosis. Primary SS with renal involvement (distal renal tubular acidosis) was diagnosed, and corticosteroid therapy with prednisolone (1 mg/kg/day) was initiated alongside continued bicarbonate supplementation (3000 mg/day).

The patient showed significant improvement, with resolution of hypokalemia and better control of glandular symptoms, and no other systemic manifestations were identified. During a nine-month follow-up period, prednisolone was gradually tapered to 2.5 mg/day. However, this dose reduction led to the recurrence of hypokalemia and sicca syndrome. Following a multidisciplinary discussion involving teams from Internal Medicine, Nephrology, and Rheumatology, prednisolone was increased to 5 mg/day, and mycophenolate mofetil (MMF) at 2000 mg/day was introduced as additional immunomodulatory therapy. Four months after initiating MMF and progressively reducing prednisolone to 2.5 mg/day alongside continued bicarbonate supplementation (3000 mg/day), the patient reported improved glandular symptoms. Nevertheless, ongoing surveillance for potential systemic involvement remains essential.

## Discussion

SS is a systemic autoimmune disorder characterized by a diverse range of autoantibodies against ubiquitous autoantigens and an autoimmune response directed at epithelial and non-epithelial structures, resulting in tissue damage and organ dysfunction [4,10]. The autoimmune basis of SS was first established in the 1960s with the discovery of specific autoantibodies like anti-Ro/SSA and SS-related antigen B antibody (anti-La/SSB) [2]. The presence of these autoantibodies, along with organ-specific lymphocyte infiltration, specifically focal lymphocytic sialadenitis in the salivary glands, is central to the pathobiology and diagnosis [2,10]. This autoimmune activity results in a wide range of clinical manifestations and dysfunction of multiple organs, including the skin, lungs, kidneys, liver, and nervous system [10].

Despite significant advances, the exact pathogenetic mechanisms underlying the immune response and self-damage in SS remain not fully understood. Current models propose that environmental triggers, such as infections or trauma, can activate the glandular epithelium, promoting an autoimmune response [10]. Viral infections are considered potential initial triggers given that the salivary gland tissues serve as locations for latent viral infections [2]. This patient’s presentation was further complicated by a recent SARS-CoV2 infection, one week prior to ED admission, a potential trigger for autoimmunity. Emerging data suggests that viral infections may exacerbate autoimmune responses, potentially accelerating disease onset. Regarding SARS-CoV2 infection specifically, it has been associated with an increased risk of new-onset autoimmune diseases, particularly within three to five months following the infection [11]. In addition to potential viral triggers, NSAIDs are a well-documented factor in inducing or exacerbating renal tubular acidosis (RTA) due to their effects on renal prostaglandin synthesis and electrolyte balance. In this patient’s case, NSAIDs were used to manage mild upper respiratory symptoms associated with SARS-CoV2 infection. While it is plausible that NSAIDs contributed to the initial worsening of RTA, the persistence of symptoms even after their discontinuation strongly suggests they were not the primary cause.

Pathologically, the clinical manifestations of SS are driven by abnormal T and B cell responses, leading to predominant lymphocytic infiltration around affected epithelial structures [2,10]. This process is often accompanied by the formation of immune complexes, particularly through cryoglobulins, resulting in chronic inflammation and progressive loss of glandular function [2]. SS is not limited to exocrine gland involvement; it can manifest systemically, affecting multiple organ systems and increasing the risk of lymphoproliferative disorders, which underscores its unique intersection between autoimmunity and lymphoproliferation [10]. 

SS is classified by presentation into primary or secondary. Primary SS occurs independently, while secondary SS manifests associated with other autoimmune conditions such as rheumatoid arthritis, systemic lupus erythematosus, dermatomyositis, or scleroderma [7,12].

Age at diagnosis is a significant factor influencing the development of systemic involvement and prognosis in SS. Studies indicate that younger patients (<35 years) tend to exhibit higher disease activity, often presenting with symptoms like fever and lymphadenopathy [13]. In the current case, the patient’s young age at diagnosis is noteworthy as it may suggest an elevated risk of complications and greater systemic disease activity.

Core symptoms of SS such as dryness of the mouth and eyes, fatigue, and joint pain affect over 80% of patients, significantly impairing their quality of life [7]. While glandular manifestations typically present as dryness, the disease's clinical spectrum can extend beyond glandular issues to encompass fatigue, Raynaud’s phenomenon, and extra-glandular manifestations involving other organs [10]. Systemic manifestations widely vary, affecting skin, lungs, liver, kidneys, and nervous system, often complicating the clinical picture with conditions like renal tubular acidosis, interstitial lung disease, and peripheral neuropathies. Notably, lymphocytic infiltration and systemic inflammation contribute to a heightened risk of lymphoma, estimated at 2-5% among SS patients [10,12]. The time between the onset of symptoms and the initial diagnosis of primary SS can extend up to 11 to 12 years [7,12], likely reflecting both the gradual advancement of the disease. The prolonged use of medications that induce dryness of mucosal surfaces, particularly antihypertensive agents, antihistamines, and antidepressants, can commonly cause a delay in recognition of sicca symptoms [2]. In the reported case, the patient had been on antidepressants for several years and reported complaints of dry mucous membranes that had persisted for approximately six months. These symptoms were attributed to the antidepressant therapy. Consequently, no further studies were conducted, despite the lack of improvement in symptoms following the substitution of antidepressant medications.

Standard diagnostic evaluation includes serological tests for ANA, anti-Ro/SSA, and anti-La/SSB antibodies, with labial salivary gland biopsy providing a histological hallmark of focal lymphocytic sialadenitis [12]. Typical laboratory results in SS are diverse: ANA is the most commonly detected (79%), while anti-Ro/SSA antibodies are identified in 73% and are recognized as the most specific [14]. Additionally, anti-La/SSB antibodies and RF are present in approximately 45-48% of the cases [14]. Some patients may exhibit cytopenia as well [10,13]. In our patient, both ANA and anti-SSA were positive, aligned with the confirmed diagnosis of SS. Diagnosis of SS is based on a weighted scoring system, with positive anti-SSA antibodies (three points) and histological evidence of focal lymphocytic sialadenitis (three points) being key components. Additional criteria include reduced tear production (Schirmer test) and unstimulated saliva flow, each contributing one point. A score ≥4 confirms primary SS [14]. In our case, the patient’s positive anti-SSA antibodies and salivary gland biopsy findings fulfilled these criteria. ANA are the most frequently detected autoantibodies in SS, with anti-Ro/SSA being the most specific, while anti-La/SSB, cryoglobulins, and hypocomplementemia stand out as prognostic markers [12].

The hallmark histological feature of SS is focal lymphocytic infiltration in the exocrine glands, typically confirmed via biopsy of the labial salivary gland or other affected glands [12]. The characteristic histopathological finding, focal lymphocytic sialadenitis, is defined by a focus score equal to greater than one per 4 mm² of tissue surface area and represents a phenotypic trait of primary SS [15,16]. Germinal centers, identified in approximately 25% of minor salivary gland biopsies, are independent predictors of glandular enlargement, systemic manifestations, and lymphoma [9,13]. Patients with a positive salivary gland biopsy at diagnosis exhibit significantly higher systemic activity during follow-up evaluations. This underscores the dual value of salivary gland biopsy as both a diagnostic and prognostic tool [13]. Even when glandular tissue appears non-pathological, it often exhibits subtle morphological changes indicative of chronic inflammation, distinguishing it from healthy tissue [17]. In our patient, the biopsy revealed the presence of germinal centers, which are associated with an increased risk of systemic manifestations, such as renal involvement in this case, and raised concerns about a potential future development of lymphoproliferative diseases, leading to closer monitoring. 

Renal involvement, although not very common (occurring in approximately 10% of cases), can lead to conditions such as tubulointerstitial nephritis (TIN) or membranoproliferative glomerulonephritis, which may result in chronic renal dysfunction with a slow progressive course [13,18]. The precise etiology and pathogenesis of renal involvement in SS remain unclear and although present in a minority of patients, it can significantly impact long-term prognosis [19]. TIN, the most common renal manifestation, typically presents insidiously, with tubular proteinuria, dRTA, and electrolyte disturbances [16,18,19]. Severe hypokalemia, a potential complication, can result in hypokalemic muscle paralysis characterized by generalized muscle weakness and, in severe cases, respiratory muscle involvement [18,19]. In the presented case, the initial presentation of the patient was hypokalemia, causing generalized muscle weakness; as the muscular involvement progressed, it eventually led to respiratory failure. Given the urgency of identifying the cause of this electrolyte disturbance, laboratory results revealing positive ANA, anti-Ro/SSA, and RF findings raised a strong suspicion of SS. This was subsequently confirmed through a glandular biopsy, which provided a definitive diagnosis. Early and timely diagnosis is crucial due to the progressive nature of renal disease in SS and the generally favorable response to treatment. Therefore, it is recommended that all patients with systemic disease manifestations undergo annual urinalysis and serum creatinine assessments. 

Management of SS has remained relatively unchanged in recent decades, with a primary focus on symptomatic relief [12]. The therapeutic approach to sicca symptoms is based on stimulation or substitution of the mechanism of the gland. This can be non-pharmacological or pharmacological, topical or systemic [12]. Systemic therapies are typically reserved for patients with active systemic disease and are tailored to the severity of symptoms and the specific organs affected. Some of the treatments consist of a combination of glucocorticoids, immunosuppressive agents, and biologic therapies such as rituximab and belimumab, particularly for patients with severe manifestations [12,13]. Although there's no cure, recent advances in biologic treatments targeting cytokines and immune pathways show promise in improving the quality of life and systemic outcomes for SS patients [14]. Therapeutic management must be personalized, addressing specific organ involvements and systemic complications to mitigate risks of progression or development of lymphoproliferative diseases like lymphoma [12]. 

The current understanding of SS continues to evolve, highlighting the importance of interdisciplinary approaches to patient management. The research underscores the need for integrating strategies to address glandular involvement with immunosuppressive therapies targeting systemic features [13]. Given the disease’s complexity and diverse manifestations, ongoing education and heightened awareness among healthcare providers are essential to achieving improved patient outcomes [12]. 

## Conclusions

This case highlights the atypical and severe manifestations of SS, including life-threatening hypokalemia and respiratory paralysis caused by distal renal tubular acidosis. Early recognition and prompt management were critical in preventing further complications. The findings, such as germinal centers in salivary gland biopsies, not only confirmed the diagnosis but also provided insight into systemic activity and potential risks.

The patient’s presentation emphasizes the importance of considering SS in cases of systemic involvement and underscores the need for a multidisciplinary approach involving rheumatology and nephrology for effective management. This case also reinforces the need for regular screening and monitoring to identify and mitigate long-term complications associated with SS, such as lymphoproliferative disorders.
